# The Utilization and Impact of Interconnected Electronic Health Record Tools on Hepatitis C Elimination Efforts in a Large Municipal Healthcare System

**DOI:** 10.3390/v17101297

**Published:** 2025-09-25

**Authors:** Eunice Casey, Kruti Gala, Gabriel Cohen, Marguerite LeLaurin, Xingyu Dai, Emma Kaplan-Lewis

**Affiliations:** New York City Health and Hospitals, Office of Ambulatory Care and Population Health, New York, NY 10004, USA; eunice.casey@nychhc.org (E.C.);

**Keywords:** hepatitis C virus, HCV, HCV elimination, universal screening, municipal health system, EHR tools

## Abstract

New York State and New York City (NYC) developed hepatitis C (HCV) elimination plans to reduce premature deaths and new infections. NYC Health + Hospitals (NYC H + H), the municipal healthcare system for NYC serving over a million individuals annually, designed electronic health record (EHR) tools that collaboratively facilitated screening, linkage, and tracking of patients diagnosed with HCV through to cure. This study reviews the impact of this group of EHR tools by comparing data on HCV testing, linkage, and cure for 12 months before tools were released and a second 12-month period coinciding with the release of tools. Indicators related to HCV screening, diagnoses, treatment initiation, and cure were assessed. All indicators reviewed improved following the implementation of EHR tools. The proportion of individuals screened increased from 34% pre-intervention to 46% during the implementation phase; the number of individuals on direct-acting antivirals increased by 11%; and the number of individuals reaching cure increased by 37%. Efforts to collaboratively develop custom interlinking EHR tools to establish a systematic process proved impactful. Integrating the needs and functions of different care settings and the structure of the local epidemic allowed for the successful development and implementation of impactful resources.

## 1. Introduction

Hepatitis C (HCV) is a communicable disease that is the leading infectious cause of cirrhosis, decompensated liver disease, and death globally. Since the advent of more effective direct-acting antivirals (DAAs) in 2011, HCV has become a curable infection with well-tolerated and relatively short (8–12 week) treatment options. Despite these advances in HCV treatment and cure, there remains a substantial population of people with uncured chronic HCV and incident HCV cases. The WHO global hepatitis strategy aims to reduce new hepatitis infections by 90% [1]. New York State (NYS) and New York City (NYC) also developed Hep C Elimination Plans [2,3]; these international, state, and city efforts all seek to reduce new HCV infections and deaths related to HCV. NYC is estimated to have 91,000 people living with chronic HCV, 40% of those not yet aware of their diagnosis [3].

NYC Health + Hospitals (NYC H + H) is the municipal healthcare system for the City of New York, servicing over 1 million individuals annually at over 70 locations across the 5 boroughs, including 11 acute care hospitals, community-based health centers, correctional health services, and long-term care facilities. In 2020, NYC H + H completed a multiyear electronic health record (EHR) transition to the Epic software platform across the acute care hospitals and community clinics of the system. The integration included using pre-programmed Epic functionality and features custom-designed by NYC H + H. In the US, over 96% of hospitals have integrated EHR systems and office-based physicians’ utilization increased from 74% in 2014 to 78% in 2021 [4]. As of 2023, Epic held 39.1% of the EHR market for acute care hospitals and 51.5% of acute multi-specialty hospitals in the US [5,6]. The Epic EHR system includes an interoperability platform, Care Everywhere, allowing for secure sharing of patient information, for individuals that consent to sharing, with other healthcare systems using Epic.

EHRs have been connected to improving quality of care through the reduction in medical errors, improved medication management, and fostering better healthcare through clinical support tools [7]. Clinical Decision Support Systems (CDSSs) are a function of EHR systems, and the most commonly used CDSSs follow a rule-based system informed by clinical practice guidelines [8]. Healthcare systems use CDSS reminders or provider alerts to improve HCV screening rates [9,10,11,12,13]; less common are interconnected EHR tools with CDSSs that support multiple steps along the HCV care continuum.

**Figure 1:**
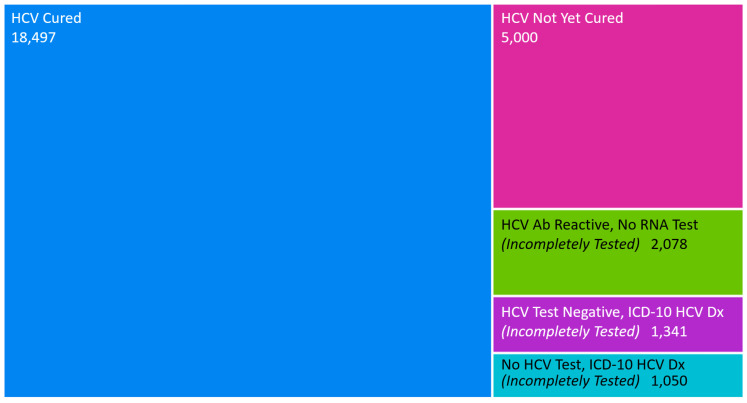
EHR Hepatitis C Registry by classification after external data integration.

Coordinated design of EHR tools with CDSS HCV efforts within NYC H + H began with the development of a custom EHR HCV Registry in December 2022, which identified 14,970 individuals. The inclusion criteria were as follows: a history of positive HCV antibody or RNA testing, or a history of a diagnosis for HCV. A classification system within the Registry was designed to identify five mutually exclusive categories to subdivide the population. The first three categories classify individuals with incomplete HCV screening: (1) no HCV test present, but an International Classification of Diseases, 10th revision (ICD-10) HCV diagnosis; (2) HCV test was negative, but an ICD-10 diagnosis; and (3) HCV Ab reactive with no RNA test. The next two categories identified (4) individuals with their last HCV RNA test on file as positive and no follow-up HCV RNA negative test, categorized as HCV diagnosed but not yet cured; and (5) individuals that have their last HCV RNA test on file as negative, categorized as HCV cured (Figure 1). The classification system is based primarily on lab data, and as such it is not possible to clarify patients that have spontaneously been cured versus those treated with DAAs. However, the system is able to track reinfection through a historic review of classifications to identify individuals previously cured but currently infected.

Upon clinical validation of the Registry and the classification system, the need to integrate external HCV lab data became apparent due to the high volume of hepatitis testing activity among the NYC H + H population in other healthcare systems. Reference to external testing was observed in a manual chart review, but was not present in structured lab data and therefore could not be used in CDSSs. Efforts were undertaken to map and integrate external HCV lab data, through the Epic Care Everywhere system, into the Registry and classification system. In January 2024, external lab data were validated and fully integrated. As a result, 4803 NYC H + H patients were newly identified as having HCV antibody positivity, current HCV infection, or cured HCV infection (Figure 1). Of the patients already in the HCV Registry, 8% (1394/17,378) had a shift in their registry classification after external data integration [14].

Focus was then placed on developing EHR tools and CDSSs that would interact with each other and the Registry and would align with and track patients through different stages of the HCV care continuum. These tools were developed in a collaborative and coordinated manner by a multi-disciplinary team of Clinical Informatics, Clinical Quality, Administration, Data Analysts, EHR programmers, HCV Patient Navigators, end-users, and clinicians.

## 2. Materials and Methods

To understand the impact of the suite of tools for HCV elimination goals and to support the ability to replicate this effort, we review the individual tools and analyze HCV elimination metrics prior to and following implementation of the tools.

NYC H + H developed and employed a suite of tools not previously available in the EHR to address HCV elimination goals. The Epic platform (Verona, WI, USA) utilized at H + H did not have an existing HCV registry and so the initial step was to create an HCV registry that incorporates internal and external lab data. Subsequently, three sets of tools were developed using the custom HCV Registry: (1) prompts to support ordering of HCV screening tests, known as Clinical Decision Support Systems (CDSSs); (2) HCV documentation supporting outreach and navigation for individuals diagnosed with HCV; and (3) an EHR-imbedded dashboard of the diagnosed population. The purpose of this combination of EHR tools is to support the coordination and tracking of patients by all members of the care team across the NYC H + H system to maximize opportunities for successful HCV intervention—be that screening, treatment, or cure support. As a group, these tools provide data related to process measures aligning with the HCV Care Continuum. To assess their effectiveness, we examine six outcome measures, collected through the HCV Registry, that related to HCV testing, diagnosis, treatment initiation, and cure. These were evaluated 12 months before the development of the coordinated tools (Pre-Intervention 1 March 2022 to 28 February 2023) compared to the 12 months coinciding with the release and integration of the EHR tools (Implementation Phase 1 March 2024–28 February 2025). These 12-month review periods are separated by 12 months corresponding to the planning and development of the EHR tools and the integration of external data in the Registry.

In summarizing the tools developed, we describe them in chronological order according to when they were built and implemented—HCV screening test CDSSs, documentation resources, and EHR-imbedded real-time dashboard. All of these tools were developed after the design and implementation of the custom HCV Registry. To develop the CDSSs, a series of rules were established to identify the target patient population that would trigger the CDSSs, indicating to an ordering provider in either Ambulatory Care, Emergency, and Inpatient care settings that the patient should be screened for HCV. HCV testing priorities followed CDC guidelines and NYS HCV testing requirements for universal once-in-a-lifetime screening for all individuals 18 and older and repeat testing for individuals of any age with increased risk for HCV infection [15,16]. The rules used to trigger HCV testing were based on the Registry classification system and indicated that either there was no history of HCV testing or that the patient fell into one of the three categories indicating incomplete testing history. In addition to the HCV Registry classifications for once-in-a-lifetime HCV screening, rules were added identifying patients at increased risk of HCV, indicating the need for annual risk-based screening due to drug-related injections and/or HIV status. Within Emergency Departments and Inpatient Units, HCV Ab testing with a reflex to RNA was automatically added to existing blood work orders if an individual met either the risk-based criteria or life-time criteria. Ordering providers have the option to decline the HCV test, but the default is to test when the criteria are met. Within Primary Care clinics, including ObGyn, the CDSSs flagged individuals for HCV screening as a health maintenance action requiring provider attention.

The second set of tools, custom HCV documentation resources, was developed collaboratively with HCV Patient Navigators. The role of the Patient Navigator is to support individuals diagnosed with HCV in connecting with an HCV care provider, identify and help them address barriers to treatment initiation, and support the patient through to cure. The documentation resources provide a structured workflow for the different processes related to this work and allow for the tracking of patients as they move from diagnosis, to care, DAA treatment initiation and through to cure. Two primary documentation tools were developed, an Outreach Form and a Navigation Form. The first supports outreach activities of diagnosed patients and the second records documentation from care initiation to cure. Both include a structured “Navigation Status” indication, enabling patient tracking once a status is selected in one of the EHR forms. There are nine Navigation Status options that identify if a patient has an appointment; has declined an appointment; is engaged in care outside of H + H; is awaiting prior authorization or patient assistance program enrollment related to DAA access; has initiated treatment, completed treatment, and if a patient has an indication of cure. The Navigation Status is updated by the Navigator at each stage of the care continuum to allow for a clear view of where an individual patient is and to support process reviews identifying disparities or opportunities for improvement at different stages of care.

**Figure 2:**
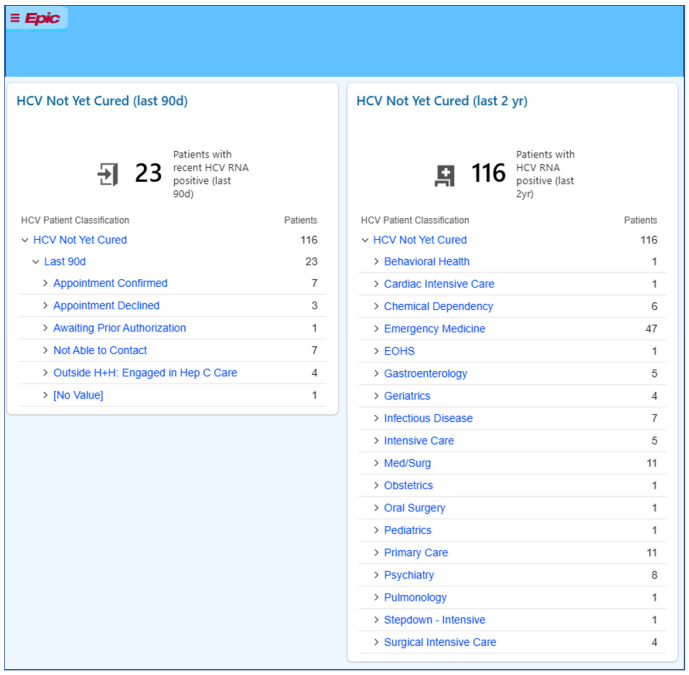
Facility-specific EHR-imbedded HCV Dashboard.

The third set of tools developed was an EHR-imbedded HCV Dashboard providing real-time data of patients testing positive for HCV RNA. The Dashboard is the central source listing all individuals in care at an individual facility that have positive HCV RNA and no indication of cure. The Dashboard includes quality data scores related to cure rate and provides two views of the population: those with a recent (last 90 days) HCV RNA test, and those with chronic infection and no indication of cure (most recent RNA test positive within the last 2 years). Individuals with HCV RNA tests within the last 90 days are divided into categories based on Navigation Status from the documentation forms, allowing for a quick understanding of which patients need either initial outreach or other navigation supports. Since the Dashboard is used by Navigators, clinicians, and administrators—all team members are able to share information and avoid duplication of effort. Figure 2 shows an example of a facility-specific view of the Dashboard with the list of patients recently screening positive and stratified by their Navigation Status on the left, and patients engaged in care with an apparent chronic HCV diagnosis (within 2 years) on the right listed by the clinic location where they receive care (e.g., Adult Medicine, ObGyn, Infectious Disease, Chemical Dependency, Methadone, etc.). This division allows for clinic-specific outreach and engagement workflows to be established, providing increased opportunities to connect patients to HCV treatment effectively. A detailed and filterable report is accessible through the dashboard, and patient charts can be opened directly from the Dashboard, allowing for care team members to review cases, access the documentation tools, and utilize additional EHR functionalities, such as group messaging and batch lab ordering to support patient care.

**Figure 3:**
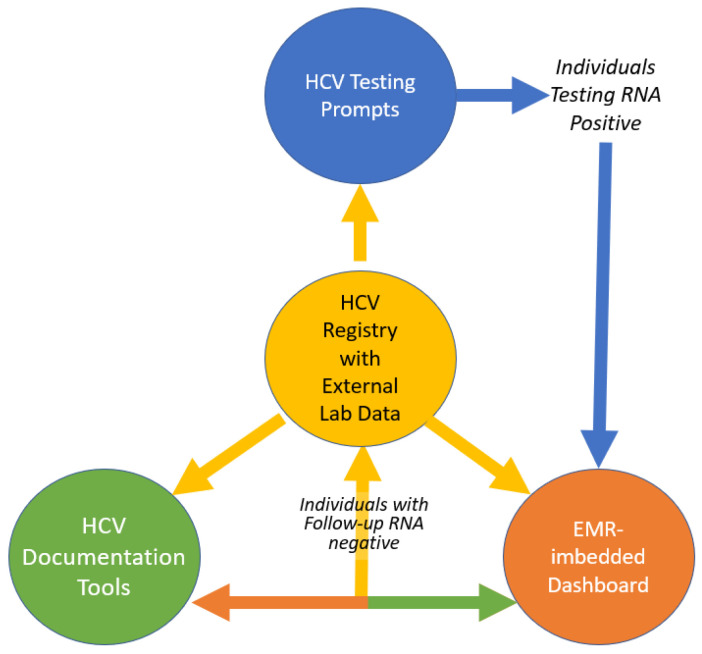
Interconnection of EHR tools.

The Dashboard and documentation tools promote coordinated care when HCV is provided in different care settings, and the HCV testing CDSSs avoid redundant testing across the healthcare system. Figure 3 demonstrates how, as individuals move through the HCV care continuum, their Navigation Status is updated, revising the Dashboard. While the lab data passively update the Dashboard, Navigators need to manually update the Navigation Status which then feeds back to the Dashboard. As patients meet clinical milestones, their category within the Registry is updated, connecting all of the tools and improving each tool’s accuracy and effectiveness.

To understand the impact, we reviewed a 12-month pre-intervention period and compared that to a 12-month period that coincided with, and followed, the implementation of the three sets of EHR tools. Indicators reviewed (Table 1) included the following: (1) the percentage of patients ever screened for HCV, defined as the number of NYC H + H patients aged 18 or older with at least one HCV test on file in the EHR before the end of the review period, among those with at least one visit during the review period; (2) the percentage of patients with no indication of HCV screening as of the review period, defined as the number of NYC H + H patients aged 18 or older receiving at least one HCV test during the report period, among those with at least one visit during the reporting period and either no HCV test on file or incomplete HCV testing based on Registry classification before the start of the review period; (3) the number of patients with confirmed active HCV infection, defined as number of NYC H + H patients aged 18 or older with HCV RNA positive result; (4) the number and proportion of patients with confirmed active HCV infection that have been prescribed DAAs, with the percentage defined as the number of NYC H + H patients aged 18 or older with DAAs prescribed in the review period among those with an RNA positive result in the review period; (5) the number of of patients ever cured, defined as the number of NYC H + H patients aged 18 or older identified as having non-reactive HCV RNA followed by reactive HCV RNA at any time; and (6) the number and proportion of patients cured within the review period, with the percentage defined as the number NYC H + H patients aged 18 or older with the last RNA test being negative, indicating “cure”, in the report period among those with an RNA positive result in the review period. The chi square difference of proportions was calculated to assess whether the changes between pre- and post-implementation phases were statistically significant.

## 3. Results

Across indicators assessing HCV screening, diagnosis, treatment, and cure, there were improvements following the integration of EHR tools (Table 1). The proportion of individuals 18 and older engaged in care at NYC H + H that met once-in-a-lifetime screening guidelines [9,10] increased from 34% to 46%. Looking only at patients eligible to receive screening during the review periods, there was an increase from 18% to 21% in the proportion of eligible individuals tested within the review period. There was a 28% increase in the volume of individuals receiving an HCV RNA positive result between the pre-intervention and implementation review periods. When looking at the impact on the connection to DAAs prescribed within NYC H + H between the two review periods, there was an 11% increase, going from 230 to 256 patients prescribed DAAs. The data system did not allow for the complete integration of external prescription data. As a result, among individuals with a positive HCV RNA result, we were able to determine that the proportion of those prescribed DAAs decreased from 20% to 17%. Despite incomplete prescription data, we observed a 156% increase in individuals who achieved “HCV Cured” (6645 patients pre-intervention to 17,015 after tool implementation), primarily related to the impact of integrating external data. Limiting the review to patients with positive RNA tests during the review period, we saw a 36% increase (198 pre-intervention to 272 after tool implementation) in the number of individuals achieving “HCV Cured” by the end of the review period, although this increase was not statistically significant. Data limitations include the inability to distinguish patients that were cured following treatment and those who were spontaneously cured of HCV. It should also be noted that Table 1 clarifies which data are from only NYC H + H and which data include external lab results, detailing the impact of services directly provided at NYC H + H separately from the improved understanding of care related to the integration of external lab data.

## 4. Discussion

To address HCV elimination goals, we sought to build interconnected tools to establish a comprehensive network of supports and resources to help patients into and through the HCV care continuum, from HCV screening and diagnosis to linkage to care, treatment, and HCV cure. Working with key stakeholders across different disciplines within the health system was foundational for successfully designing and implementing EHR tools. This effort included working with clinical and administrative leads to review clinical workflows and map different clinical referral locations for HCV treatment (e.g., Primary Care, Infectious Disease, Gastroenterology); working with IT specialists to develop and build EHR tools; collaborating with navigation staff to understand resources for patient outreach/navigation and tracking; and collaborating with front-line staff to achieve communication and education on the tools.

Others have published research on the efficacy of EHR prompts for increasing HCV screening [4,5,6]; however, to our knowledge, this is the first published report of a coordinated EHR tool approach to address HCV elimination goals across a healthcare system. While discrete EHR prompts can be helpful, the interconnection of tools addressing different components of the HCV care continuum was key to the success of this intervention. Starting with an HCV Registry that established clinically relevant classification based on the HCV care continuum, the effectiveness of that Registry expanded through the incorporation of external HCV lab data. Using that foundation, we built HCV CDSS testing prompts designed to work effectively within specific care settings and established a real-time EHR-imbedded Dashboard and documentation tools that worked together to support patient outreach and tracking by front-line teams, allowing for coordinated care.

Oftentimes, initiatives to improve screening or treatment of a communicable disease are implemented in silos, which hinders the potential for broader success and can contribute to fragmentation of care or augment cracks in the system that individuals can slip through [17,18]. Maximizing the opportunities provided by public health campaigns such as HCV elimination requires a systems-level approach that recognizes in its design [19] how people seek healthcare—specifically across many institutions, departments, and care settings.

The limitations of this study include a relatively short period of follow-up, the fact that external data mapping does not include all NYC healthcare institutions, and the fact that it is limited to laboratory values, minimizing the ability to understand treatment initiation outside of the NYC H + H system. Despite these limitations, the coordinated series of tools demonstrated high success at increasing rates of HCV screening, diagnosis, linkage to care, treatment initiation, and ultimately cure across a large municipal healthcare system.

## 5. Conclusions

HCV elimination is possible due to the advent of well-tolerated and relatively short treatment options. To maximize this opportunity, there is a need for a coordinated response within healthcare systems to effectively screen and diagnose individuals, connect them to treatment, and support them through to cure. Healthcare system EHRs provide potent resources that can be harnessed to support all steps along the HCV care continuum. Tools should be interconnected and developed to maximize impact, avoid gaps, and reduce redundancies. A multi-disciplinary team with stakeholders across all areas of HCV care, including front-line clinical care teams, administrators, and IT experts, is instrumental in designing and successfully implementing valuable and effective tools. EHR tools can be even more potent when created in this manner and can be valuable assets in campaigns to end epidemics.

## Figures and Tables

**Table 1:** Change in rates of HCV screening, diagnosis, treatment, and cure after EHR tool implementation.

	Pre-Intervention 1 March 2022–28 February 2023		Implementation Phase 1 March 2024–28 February 2025		Chi Square Test
% of patients ever screened for HCV	34% (212,513/619,210) *Data Source: NYC H + H Lab*		46% (311,912/682,300) *Data Source: NYC H + H Lab and external labs*		*p*-value < 0.000
% of patients with no indication of HCV testing as of review period	18% (88,765/495,467) *Data Source: NYC H + H Lab*		21% (89,052/422,427) *Data Source: NYC H + H Lab and external labs*		*p*-value < 0.000
# of patients with confirmed HCV infection	1164 *Data Source: NYC H + H Lab*		1502 *Data Source: NYC H + H Lab and external labs*		
% and # of patients with confirmed HCV infection prescribed DAAs	*Number* 230	*Rate* 19.7% (230/1164)	*Number* 256	*Rate* Rate: 17% (256/1502)	*p*-value = 0.072
	*Data Source: NYC H + H Lab*		*Data Source: NYC H + H Lab and external labs*		
# of patients having ever achieved “HCV Cured”	6645 *Data Source: NYC H + H Lab*		17,015 *Data Source: NYC H + H Lab and external labs*		
% and # of patients having achieved “HCV Cured” within review period	*Number* 198	*Rate* 17% (198/1164)	*Number* 272	*Rate* 18.1% (272/1502)	*p*-value = 0.460
	*Data Source: NYC H + H Lab*		*Data Source: NYC H + H Lab and* *external labs*		

## Data Availability

The original contributions presented in this study are included in the article. Further inquiries can be directed to the corresponding author(s).
